# Cannabinoid Receptor 1 Expression in Human Dorsal Root Ganglia and CB13-Induced Bidirectional Modulation of Sensory Neuron Activity

**DOI:** 10.3389/fpain.2021.721332

**Published:** 2021-11-16

**Authors:** Zachary K. Ford, Ashlie N. Reker, Sisi Chen, Feni Kadakia, Alexander Bunk, Steve Davidson

**Affiliations:** ^1^Neuroscience Graduate Program, College of Medicine, University of Cincinnati, Cincinnati, OH, United States; ^2^Department of Anesthesiology and Pain Research Center, University of Cincinnati, College of Medicine, Cincinnati, OH, United States

**Keywords:** pain, cannabinoid, dorsal root ganglia, inflammation, TRPV1, sensory neuron

## Abstract

Cannabinoid receptors have been identified as potential targets for analgesia from studies on animal physiology and behavior, and from human clinical trials. Here, we sought to improve translational understanding of the mechanisms of cannabinoid-mediated peripheral analgesia. Human lumbar dorsal root ganglia were rapidly recovered from organ donors to perform physiological and anatomical investigations into the potential for cannabinoids to mediate analgesia at the level of the peripheral nervous system. Anatomical characterization of *in situ* gene expression and immunoreactivity showed that 61 and 53% of human sensory neurons express the CB1 gene and receptor, respectively. Calcium influx evoked by the algogen capsaicin was measured by Fura-2AM in dissociated human sensory neurons pre-exposed to the inflammatory mediator prostaglandin E2 (PGE2) alone or together with CB13 (1 μM), a cannabinoid agonist with limited blood–brain barrier permeability. Both a higher proportion of neurons and a greater magnitude of response to capsaicin were observed after exposure to CB13, indicating cannabinoid-mediated sensitization. In contrast, membrane properties measured by patch-clamp electrophysiology demonstrated that CB13 suppressed excitability and reduced action potential discharge in PGE2-pre-incubated sensory neurons, suggesting the suppression of sensitization. This bidirectional modulation of sensory neuron activity suggests that cannabinoids may suppress overall membrane excitability while simultaneously enhancing responsivity to TRPV1-mediated stimuli. We conclude that peripherally restricted cannabinoids may have both pro- and anti-nociceptive effects in human sensory neurons.

## Introduction

Some clinical studies have noted analgesic benefits of cannabinoids for various chronic pain syndromes, including neuropathic, rheumatoid arthritis, and cancer pain (1–4). However, the overall assessment of benefit of cannabinoid use for pain patients remains inconclusive (5, 6), and concerns over increased levels of treatment-emergent adverse events compromise general acceptance of medicinal use (7). Cannabinoid receptors are highly expressed within nearly all parts of the nervous system, including within cognitive, affective, and motivational systems, and therefore, cannabinoid ligands can lead to unwanted side effects such as psychoactivity, dependence, and sedation (8). These secondary effects can also make measurement and determination of the specific anti-nociceptive effects of systemic cannabinoids difficult to assess.

Cannabinoids bind CB1 and CB2 G-protein-coupled receptors, and while sequence homology between these two receptor is low, both canonically signal *via* Gi/o to inhibit intracellular adenylyl cyclase production (9). Cannabinoid receptors are found in rodent peripheral nervous system, in the dorsal root ganglia, and the CB1 receptor subtype is localized to rodent sensory neurons, including nociceptors (10–14). Based on anatomical expression and predicted inhibitory profile, CB1 has been hypothesized to suppress neural excitability in the peripheral nervous system and reduce nociceptive signaling. Indeed, animal studies have shown that the activation of CB receptors modulates peripheral neural excitability and sensitization consistent with an anti-nociceptive phenotype. For example, in cultured rodent DRG neurons, cannabinoids inhibit acid-sensing ion channels (15) and reduce voltage-activated calcium currents (16). Cannabinoids, likewise, suppress action potentials in nociceptive c-fibers (17).

Peripheral mechanisms are thought to explain the results of myriad animal models of pain in which cannabinoid activation has been shown to exhibit analgesic properties (18, 19). For instance, knockout of CB1 from nociceptive neurons in a rodent model substantially reduced cannabinoid-mediated analgesia from systemic administration, indicating a significant role for peripheral CB1 in pain relief from cannabinoids (20). Despite animal behavior and physiology as well as some clinical human evidence supporting an analgesic role for cannabinoids, a number of human clinical trials have failed to demonstrate clear efficacy (21). As with many therapeutic models where success in animals does not always predict translation to human (22, 23), cannabinoid systems may exhibit biological differences between species. We developed strategies to recover and functionally test human sensory neurons *in vitro* and previously found that other Gi/o receptors can prevent the development of hypersensitivity from inflammatory mediators (24, 25). Here, we assess cannabinoid receptor expression and function on human sensory neurons under inflammatory conditions to better understand peripheral analgesic potential.

## Materials and Methods

### Tissue Recovery and DRG Processing

Fully consented, anonymized transplant organ donors were managed by LifeCenter, Cincinnati, and experiments were performed under University of Cincinnati IRB Study ID#: 2015-5302. Five donors contributed to the experiments: male 36 years, male 28 years, female 38 years, female 23 years, and female 16 years. All were Caucasian with body mass index between 24 and 33. Protocol for the recovery of human DRG was as described (26). Briefly, at the University of Cincinnati Medical Center, after the recovery of transplant organs and within 90 min of aortic cross-clamp, peripheral nerves, DRGs, and roots were recovered and stored in ice-cold *N*-methyl-D-glucamine (NMDG) artificial spinal fluid (aCSF) (in mM: 93 NMDG, 2.5 KCl, 1.25 NaH_2_PO_4_, 30 NaHCO_3_, 20 HEPES, 25 glucose, 5 ascorbic acid, 2 thiourea, 3 Na^+^ pyruvate, 10 MgSO_4_, 0.5 CaCl_2_, 12 N-acetylcysteine, and 4.24 ml 10N HCl, pH 7.4). Tissues were then immediately transported to the laboratory where connective tissue, fat, and dura mater were removed and nerve and roots of L3 and L4 ganglia trimmed in ice-cold NMDG-aCSF.

For *in situ* hybridization (ISH) and immunohistochemistry (IHC), hDRG was blocked and fixed overnight at 4°C in 4% paraformaldehyde in PBS (pH 7.4), then transferred to 30% sucrose overnight at 4°C. Tissue was embedded in Tissue Plus (Fisher Healthcare), cryosectioned, and stored at −80°C.

For cell culture, the ganglia were diced and then incubated in papain in HBSS+ 10 mM HEPES (Gibco), for 40 min at 37°C, 5% CO_2_ followed by a 40-min incubation with collagenase (Sigma, St. Louis, MO) and then put into Neurobasal-A DRG media containing [penicillin/streptomycin, B27 supplement, GlutaMAX, FBS (ThermoFisher/Gibco)]. Dissociated DRG were filtered through a 100-μm filter and then plated onto poly-d-lysine/collagen (Sigma)-coated cover slips and incubated for 2–4 days for use in calcium imaging and electrophysiological studies.

### *In situ* Hybridization and IHC

*In situ* hybridization was performed using RNAscope (Advanced Cell Diagnostics Inc.; Newark, CA). Fixation and pretreatment steps were carried out as per manufacturer's instructions using fresh-frozen 14-μm hDRG sections. The Hs-CBR1 RNAscope probe (591521) was hybridized and developed using the RNAscope Multiplex Fluorescent v2. TSA Plus Cyanine 5 systems (1:1,000, PerkinElmer) was diluted with TSA buffer to visualize the CBR1 signal. Slides were washed, counterstained with DAPI from the RNAscope kit, and cover-slipped with Prolong Gold Antifade Reagent (Invitrogen, Eugene, OR).

For IHC, tissue sections or PFA-fixed cells were washed with 0.025% Triton X-100 in PBS followed by incubation in blocking serum for 1 h (Superblocker Fisher; Rockford, IL), and then incubated overnight at 4°C with a polyclonal rabbit anti-CB1 antibody (1:100; code ab23703; Abcam, Cambridge, UK). Sections or cells were then incubated for 1 h at room temperature with donkey anti-rabbit Alexa 555 (1:1,000; code A31572; Life Technologies, Eugene, OR) and counterstained with DAPI (4′,6-diamidino-2-phenylindole).

Images were captured on the BZ-X810 fluorescence microscope (Keyence, Osaka, Japan). Images of the full hDRG were taken *via* 10× z-stacks and stitched together using the BZ-8000 analyzer software. 20× z-stack images were also obtained. Cell counts were performed manually using the ImageJ (NIH, Bethesda) multipoint count feature. Neurons were identified by the ring of DAPI-containing satellite glia. For RNAscope, neurons with a minimum of five or more fluorescent puncta were counted as a CB1R positive. For IHC quantification, CB1-immunoreactive neurons were detected by Olympus CellSens software (Olympus, Tokyo, Japan) through 555-nm channel and illumination intensity threshold functions and filtered by area. Cell counts were averaged from two sections separated by at least 150 μm per DRG, then averaged across three separate donors.

### Calcium Imaging

Dissociated hDRG were incubated with Fura2-AM (3 μg/ml, ThermoFisher) for approximately 45 min, then transferred to a recording chamber in external recording solution (in mM): 130 NaCl, 5 KCl, 2 CaCl_2_, 1 MgCl_2_, 30 glucose, and 10 HEPES. Prostaglandin E2, 1 μM (Sigma), and cannabinoid agonist CB13, 1 μM (Tocris), were applied either 20 min prior to transfer (pre-incubation) or bath-applied for 3 min in between a first and second pulse of 250 nM capsaicin (Sigma) followed by 60 mM KCl (Sigma). Illuminance signal was acquired using 365/385-nm switching LED (pE4000, CoolLED) controlled by MetaFluor software (Molecular Devices) on an Olympus BX51 microscope. Response intensity was calculated by taking the peak Fura-2 signal against a baseline measure immediately prior to each stimulus. A responder was counted as any cell that increased ΔF signal at least a 5% on the first, or 3% on the second capsaicin pulse, and produced a response to 60 mM KCl. Cells that did not respond either to capsaicin or to KCl were eliminated from the analysis.

### Electrophysiology

Neurons were recorded in an external recording solution consisting of (in mM): 145 NaCl, 3 KCl, 2.5 CaCl_2_, 1.2 MgCl_2_, 7 glucose, and 10 HEPES, adjusted to pH 7.4 with NaOH and 305 mOsm with sucrose. Borosilicate, filamented glass electrodes with 2–4 MΩ resistance (Warner Instruments, Hamden, CT) contained internal solution (in mM): 130 K-gluconate, 5 KCl, 5 NaCl, 3 Mg-ATP, 0.3 EGTA, and 10 HEPES, adjusted to pH 7.3 with KOH and 294 mOsm with sucrose. Liquid junction potential was zeroed. After establishing gigaseal and then break-in, a series of protocols were executed to characterize the membrane excitability (24). PGE_2_ (1 μM) with or without CB13 (1 μM) was incubated with hDRG 20 min prior to recordings. Rheobase was established from the square current step at which the first action potential was triggered. The first action potential of a train was used to determine the threshold, defined as the voltage at which the first derivative of the membrane potential increased by 10 V/s. Neurons were viewed on an Olympus BX-50 epifluorescence microscope. Recordings were obtained on a MultiClamp 700B and sampled at 20 kHz and filtered at 10 kHz using a Digidata 1550A (Molecular Devices). Data were collected and analyzed with Clampex 10.5 and Clampfit 10.5 software (Molecular Devices). Series resistance was kept below 10 MΩ in all recordings, and only cells with a diameter of ≤60 μm were studied.

### Data Analysis

All statistical analyses were performed using GraphPad Prism 7. Calcium imaging magnitude comparisons were done using a one-way ANOVA with Dunnett's post-test, while responder counts were done using a χ^2^ test. Electrophysiological data were analyzed using either two-way ANOVA or one-way ANOVA followed by Tukey's multiple comparisons post-test. Significance was defined as *p* < 0.05. Data are presented as mean ± SEM.

## Results

### Human Primary Sensory Neurons Express CB1 Receptor

**Figure 1:**
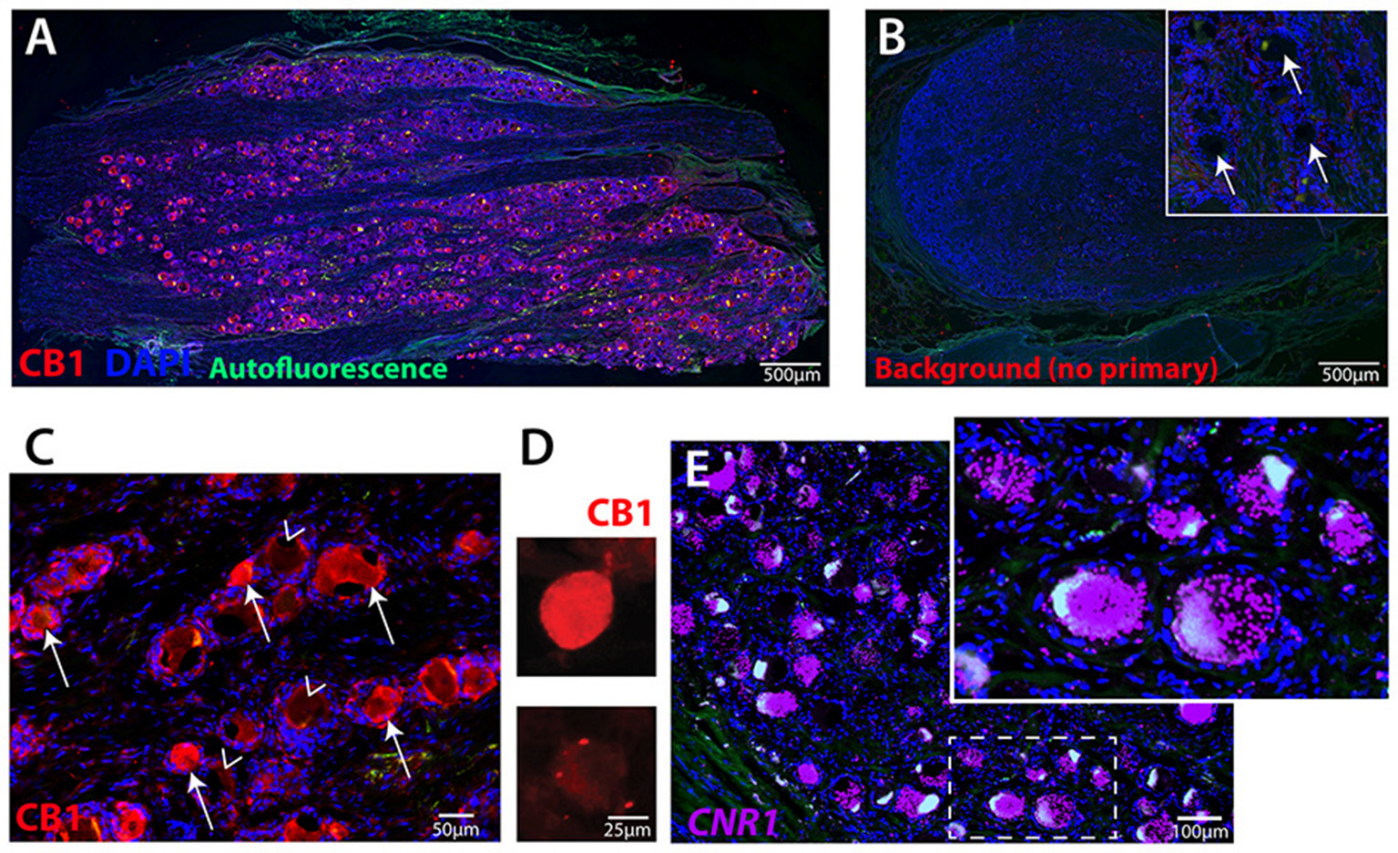
Human primary sensory neurons robustly express CB1 receptor. **(A)** Image of hDRG section showing CB1 expression (red), DAPI nuclear expression (blue), autofluorescence (green). **(B)** hDRG section with DAPI and no primary antibody showing the nuclei of satellite cells encircling neuron soma locations (inset, arrows). **(C)** CB1 primary antibody and alexa-fluor 555 secondary indicates neuronal expression of CB1 receptor protein on many (arrows), but not all (arrowheads), neurons. **(D)** Dissociated hDRG neurons in culture retain CB1 expression detected by fluorescent secondary, and **(E)** Fluorescent *in situ* hybridization (RNAscope) for human CNR1 gene. Inset shows highly-labeled cells with densely packed puncta.

We examined the expression of the CB1 receptor from IHC images of lumbar DRG from two male donors and one female donor. Quantification revealed that 53 ± 5.1% of lumbar human sensory neurons expressed immunofluorescence for CB1R (Figures 1A–C). As is typical, a high level of autofluorescence was observed in hDRG, as was the buildup of lipofuscin. A no primary antibody control with DAPI shows that the location of sensory neurons is easily observed, encircled within an annulus of putative satellite glia cells (Figure 1B, inset). Higher magnification image of CB1 primary antibody revealed cytoplasmic CB1-immunoreactivity in sensory neurons (Figure 1C). Expression of CB1 was also observed in dissociated sensory neurons fixed after 3 days in culture using fluorescent-tagged secondary immunochemistry (Figure 1D). To characterize the expression pattern of the CB1 receptor gene, *cnr1*, in human dorsal root ganglia, we performed RNA scope (Figure 1E). Expression was robust and showed a large number of both large- and small-sized neurons with densely packed puncta. Quantification of *cnr1* revealed that 62 ± 7.6% of neurons were positive across the same three donors used for quantification of the IHC experiments. Together, these data demonstrate the widespread expression of CB1 receptor gene and protein product in human sensory neurons that is maintained in culture.

### CB13 Suppressed Membrane Excitability in PGE_2_-Incubated HDRG Neurons

**Figure 2:**
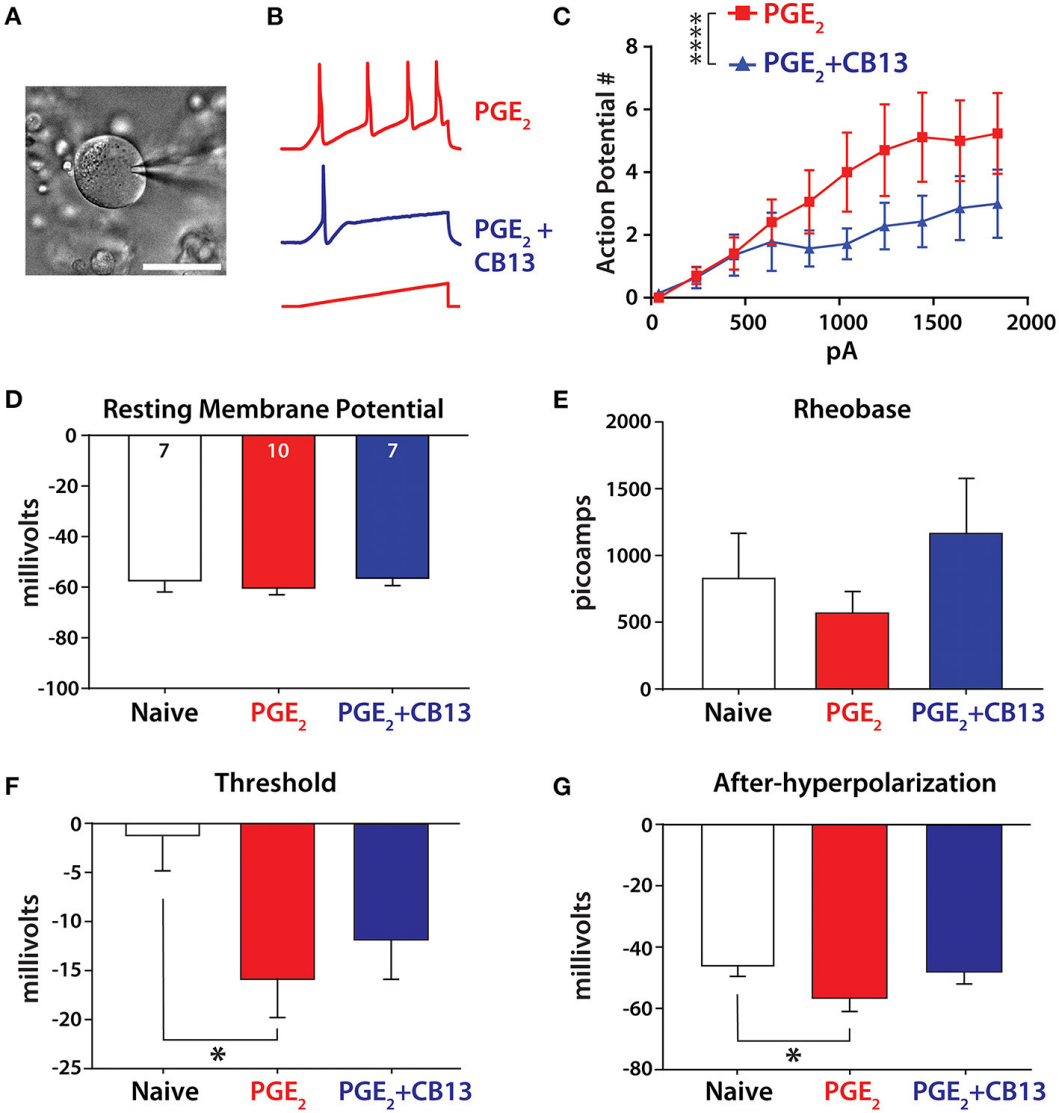
CB13 reduces PGE_2_-induced sensitization of primary sensory neurons. **(A)** Image of hDRG neuron under patch clamp. Scale bar = 50 μm. **(B)** Example traces from PGE_2_ and PGE_2_+CB13-treated cells showing action potential discharge. A 500-ms ramp current protocol increased 200 pA in each step. **(C)** Action potential discharge from stepwise increasing current ramp. Two-way ANOVA, *p* < 0.0001. **(D)** Resting membrane potential, cell numbers inset. **(E)** Rheobase determined as the minimum current applied as a 800-ms square pulse-stepped 100 pA per cycle to elicit an action potential. **(F)** Threshold was taken as the point where the first derivative of voltage exceeded 10 V/s on the first action potential from a ramp stimulus. One-way ANOVA with Tukey's multiple comparisons, *p* < 0.05. **(G)** After-hyperpolarization was the most negative voltage reached after an action potential was elicited from a short, 2-ms step protocol. One-way ANOVA with Tukey's multiple comparisons, *p < 0.05; ****p < 0.0001.

To detect if the cannabinoid agonist CB13 could alter the membrane excitability of hDRG neurons, neurons were patched in current clamp mode to examine response properties to current injection under incubation with PGE_2_ or PGE_2_ + CB13 (all at 1 μM). The addition of CB13 significantly reduced the number of action potentials (Figures 2A–C). We next tested membrane properties and observed no changes from the different treatment groups to resting membrane potential (Figure 2D) or the amount of current required to elicit the first action potential (rheobase, Figure 2E). However, PGE_2_ significantly lowered the membrane voltage threshold to trigger an action potential compared to untreated cells, but this was mitigated by CB13 (Figure 2F). Likewise, the nadir of membrane voltage reached after an action potential (after-hyperpolarization) was significantly more negative with PGE_2_ compared to untreated neurons, and again this was mitigated by CB13 (Figure 2G). These data indicate that PGE_2_ sensitizes hDRG neurons, consistent with our previous studies (24, 25), and that CB13 reduces the effects of PGE_2_.

### CB13 and PGE_2_ Interact to Generate Sensitization to Capsaicin

**Figure 3:**
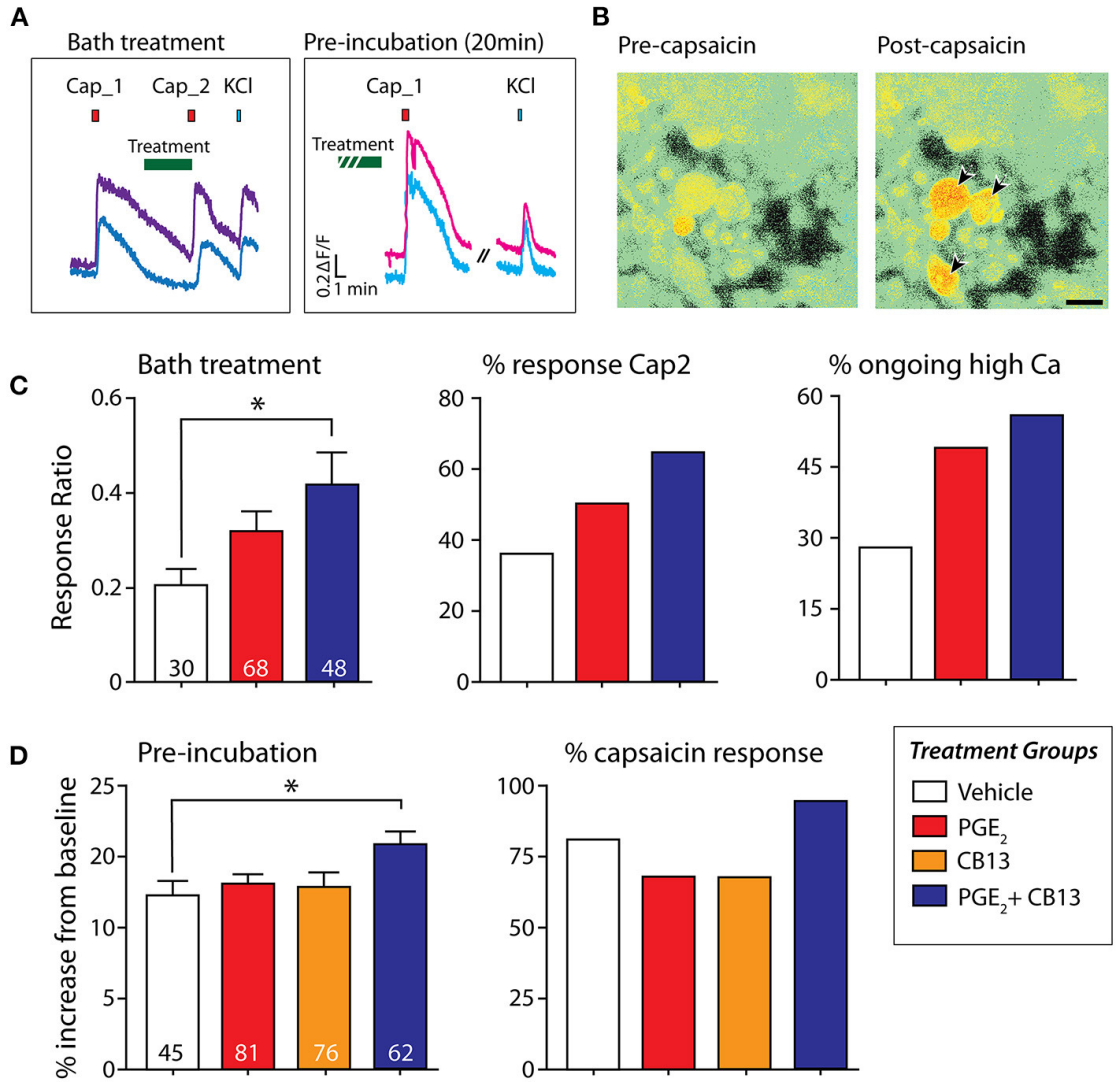
CB13 and PGE_2_ interact to enhance sensitization to capsaicin. **(A)** Example of “bath treatment,” in which treatment is bath-applied in between capsaicin applications, and “pre-incubation,” in which dissociated hDRG are exposed to treatment under incubator conditions for 20 min prior to capsaicin. **(B)** Fura-2AM signal in hDRG neurons showing the response in some cells to 250 nM capsaicin (arrows). **(C)** Bath treatment: Cap2/Cap1 ratio (*left*) one-way ANOVA followed by Dunnett's post-test shows that PGE_2_+CB13 leads to significantly reduced desensitization (*p* = 0.02). Number of cells tested in each group indicated at the bottom of each bar. *Center*, Percent of cells responding to the second capsaicin application. *Right*, Percent of cells that did not fall back to baseline within 7 min after the first capsaicin application. **(D)** Pre-incubation: (*Left*) magnitude of peak capsaicin response. One-way ANOVA followed by Dunnett's post-test shows PGE_2_ + CB13 exhibited a larger peak response compared to vehicle. (*Right*) Percent of sensory neurons responsive to capsaicin after 20 min of incubation with the various treatments. *p < 0.05.

To determine the effects that CB13 may have on the nociceptive heat and acid-sensitive channel transient receptor potential vanilloid 1 (TRPV1) under inflammatory conditions, we monitored intracellular calcium responses to 250 nM capsaicin following the treatment with PGE_2_, PGE_2_ + CB13, or CB13 alone. Dissociated neurons were either bath-treated in between the first and second capsaicin pulses, or pre-incubated with treatment for 20 min (Figure 3A). Capsaicin responses were monitored by calcium indicator Fura-2AM (Figure 3B). When PGE_2_ + CB13 treatment was bath-applied for 2 min after an initial capsaicin pulse, the magnitude of response of the second capsaicin pulse relative to the first (sensitization ratio) was significantly increased by PGE_2_ + CB13, indicating reduced desensitization (Figure 3C). Similarly, the proportion of neurons responding to the second pulse of capsaicin was highest in the PGE_2_ + CB13 group (Figure 3C). In a separate set of experiments, cells were pre-incubated for 20 min with PGE_2_ alone or PGE_2_ + CB13 treatment conditions, and then capsaicin was bath-applied. Under these conditions, a significant increase in the magnitude of peak calcium in response to capsaicin was observed in the group treated with both PGE_2_ + CB13 compared with control. The proportion of neurons responding to capsaicin was also highest in the PGE_2_ + CB13 group (Figure 3D).

## Discussion

Given the societal need but lagging pace of discovery for safe and efficacious analgesics, concern has arisen over the perceived obstacle of significant species differences between rodent models and humans. This has led to an acceleration of research effort aimed at investigating human tissues directly (27). Unprecedented recent characterization of human dorsal root ganglia through gene analysis (28) and physiological studies with live human sensory neurons *in vitro* (29–32) have begun to draw comparisons and some contrasts with the rodent literature.

**Figure 4:**
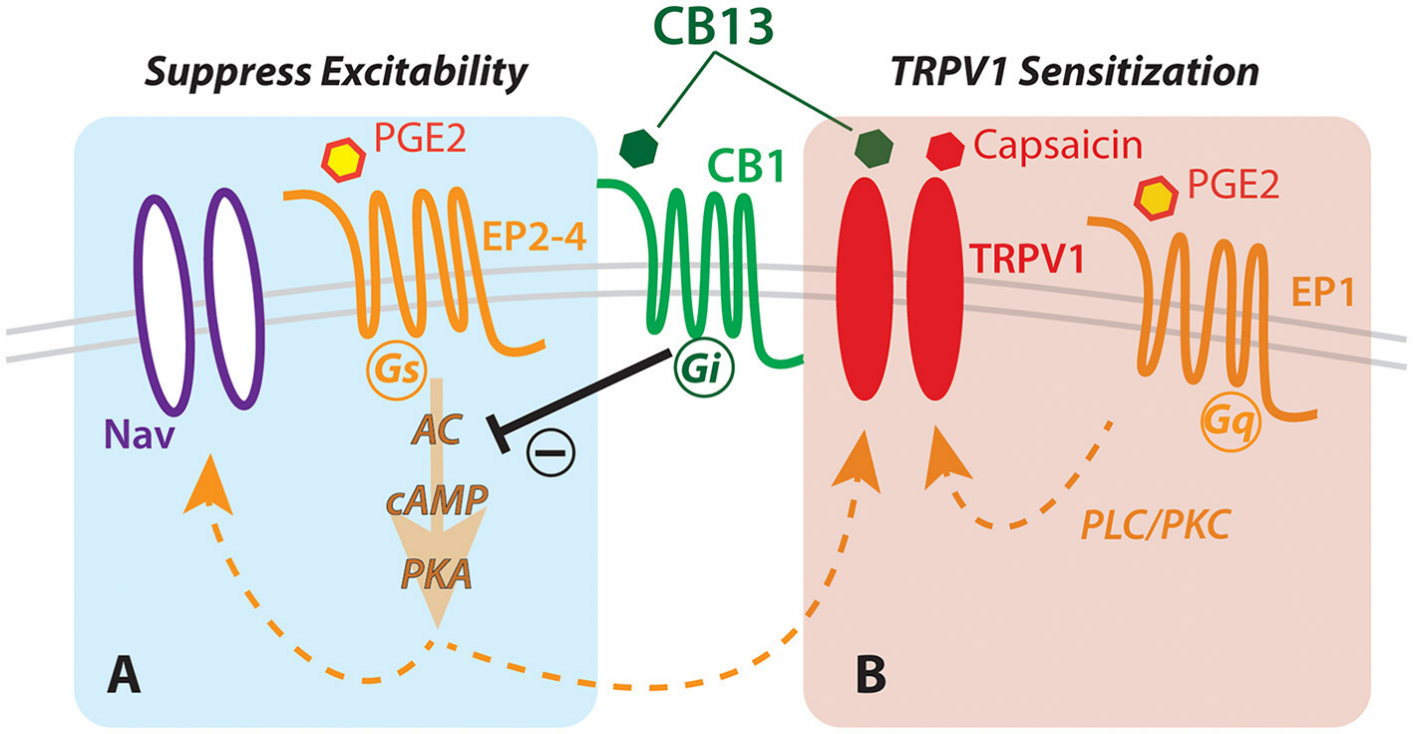
Hypothesized schematic of cannabinoid interaction with TRPV1 and EP receptors in human sensory neurons. Dual actions of cannabinoid (CB13) on the CB1 receptor leading to **(A)** G-protein-coupled receptor-mediated inhibition of cAMP production *via* prostaglandin receptor, and **(B)** direct action on TRPV1 leading to enhanced sensitization. *Nav, voltage-gated sodium channels; PKA, protein kinase A; cAMP, cyclic AMP; AC, adenylyl cyclase; EP1-4, prostaglandin receptor subtypes; PLC/PKC, phospholipase/protein kinase C*.

In this study, we examined the expression of cannabinoid receptor type 1 protein and gene in human dorsal root ganglia and found it to be expressed abundantly in many sensory neurons, both large and small. Following from this anatomical evidence, we considered the possibility that cannabinoids could modulate the neural activity with implications for nociception. We found that the cannabinoid agonist CB13 could suppress membrane hyperexcitability in live human sensory neurons exposed to the inflammatory mediator PGE2. In contrast, however, CB13 also enhanced sensitization of the capsaicin response under inflammatory conditions. These bidirectional effects with regard to the potential for analgesia should be carefully considered as cannabinoids are further investigated or even administered in clinical settings (Figure 4).

The CB1/CB2 agonist, CB13 [also known as SAB378 or naphthalen-1-yl-(4-pentyloxynaphthalen-1-yl)methanone], is functional at human CB1 and CB2 receptors and possesses limited blood–brain barrier permeability, keeping it restricted from the central nervous system (33). A phase 1 study demonstrated the oral bioavailability of CB13 in humans (34). Anti-hyperalgesic effects were reported when CB13 was given orally *in vivo* in a rodent neuropathic pain model (33). CB13 also reduced inflammation in a rodent colitis model (35) and produced long-lasting analgesia in a rodent chronic constriction injury model of neuropathic pain when synthesized with nanoparticles (36). These studies indicate low toxicity and good bioavailability of CB13 in human and analgesic potential in animal models. Because it is peripherally restricted, centrally mediated unwanted secondary effects could be limited, making CB13 or a similar derivative clinically attractive. Therefore, we used CB13 to test the modulation of neural activity in human peripheral sensory neurons.

PGE_2_ is potent endogenous mediator of pain and inflammation (37). Hypersensitivity from PGE_2_ is mediated in part by enhancing the function of the capsaicin and heat receptor, TRPV1 (38), and also modulating voltage-gated ion channels such as the tetrodotoxin-resistant sodium channels in nociceptors (39). In dissociated human sensory neurons, PGE_2_ produced overt action potential discharge in some cells and hyperexcitability (24). In this study, we found that 1 μM PGE_2_ modestly sensitized sensory neurons when applied for 20 min as a pretreatment and that co-administration of 1 μM CB13 blocked this sensitization. This result is consistent with our earlier finding that another inhibitory G-protein-coupled receptor, the group II metabotropic glutamate receptor, blocked PGE_2_ sensitization on human sensory neurons (25). These data support idea that cannabinoids can act as an analgesic to reduce enhanced nociception at the periphery caused by inflammation.

To more directly assess the possibility that cannabinoids regulate TRPV1, we used calcium imaging to examine responses to capsaicin under inflammatory conditions with PGE_2._ Somewhat unexpectedly, in both pre-incubation and bath-applied groups, 1 μM PGE_2_ alone did not elicit a significant increase in either the magnitude of response or the number of responding cells to 250 nM capsaicin. Previous calcium imaging experiments were successful in establishing significant PGE_2_ enhanced responses to capsaicin in hDRG (40). Although we observed trends in the same direction in our dataset, we may have been underpowered relative to the Sheahan et al.'s (40) study to detect small differences between means as significant due to fewer number of neurons live-imaged for this experiment—a consequence of limited donor availability. Nevertheless, cells that were treated with both PGE_2_ and CB13 surprisingly did show a significantly higher ratio of capsaicin responders and produced an increase in the magnitude of capsaicin responses when compared with vehicle-treated cells.

Although CB13 is not recognized as a TRPV1 agonist and lacks structural similarity with capsaicin, one of the best-studied endocannabinoids, arachidonylethanolamide (anandamide), also functions as an endovanillioid, and binds to and activates TRPV1 *in vitro* and produces a TRPV1-mediated vasodilative effect *in vivo* (41). Other endocannabinoids, also vanilloid-like long-chain fatty acid molecules, were subsequently found to act on TRPV1, including n-arachidonyl-dopamine (NADA), which behaves as a full TRPV1 agonist (42, 43), and 2-arachidonyl glyceryl ether, which acts on exogenously expressed TRPV1 and produces non-CB1/2-mediated vasodilative effects in rats (44). The effective concentration of anandamide to activate TRPV1 is approximately 100-fold greater than capsaicin (41, 45); this may explain why we never observed any direct Ca^2+^ responses to 1 μM concentration of CB13. On the other hand, when CB13 was applied with PGE_2_, there was a greater response to capsaicin than with either compound alone. It is worth noting that the relationship between cannabinoids and TRPV1 appears to be complex, and several investigations have contrarily shown that CB1 agonists can block some forms of TRPV1 sensitization (46, 47).

The canonical effect of CB13 on CB receptors is through Gi/o-mediated inhibition of adenylyl cyclase, the stimulatory target of Gs-coupled prostaglandin receptors EP2,4 (48). Inhibition of adenylyl cyclase lowers intracellular cAMP and PKA, and consequently, it reduces phosphorylation and sensitization of TRPV1 (49). However, another prostaglandin receptor, the Gq-coupled EP1, expressed in human sensory neurons (50), activates phospholipase C (PLC) and protein kinase C (PKC) (48, 51). PKC (like PKA) also phosphorylates TRPV1 and sensitizes TRPV1 to anandamide and capsaicin (52, 53). Therefore, despite any potential inhibitory effect of CB13 on TRPV1 through CB1-mediated PKA inhibition, the activation of EP1 by PGE_2_ may also trigger an alternative pathway through PKC to sensitize TRPV1 to capsaicin in a manner resistant to CB1/2-mediated inhibition of adenylyl cyclase (Figure 4).

Of course, the failure of CB13 to prevent sensitization of TRPV1, and instead enhance TRPV1 responses, does not support the efficacy of CB13 as a broad analgesic. Yet, the electrophysiological data provide compelling evidence that cannabinoids are capable of suppressing human sensory neuron excitability, including action potentials, which is the principal means of signaling nociception to the spinal cord. Therefore, cannabinoid agonists have the potential to reduce pain at the periphery, away from confounding central effects, although novel pharmacological development may be required to mitigate hypersensitivity of TRPV1 caused by the current generation of cannabinoid agonists.

## Data Availability Statement

The raw data supporting the conclusions of this article will be made available by the authors, without undue reservation.

## Author Contributions

ZF, AR, SC, FK, and SD designed experiments. ZF, AR, SC, SD, AB, and FK conducted experiments and analyzed data. ZF, AR, AB, and SD wrote manuscript. All authors contributed to the article and approved the submitted version.

## Funding

This work was supported by NIH/NINDS (Grant Nos. RF1NS113881 and R01NS107356).

## Conflict of Interest

The authors declare that the research was conducted in the absence of any commercial or financial relationships that could be construed as a potential conflict of interest.

## Publisher's Note

All claims expressed in this article are solely those of the authors and do not necessarily represent those of their affiliated organizations, or those of the publisher, the editors and the reviewers. Any product that may be evaluated in this article, or claim that may be made by its manufacturer, is not guaranteed or endorsed by the publisher.
